# Supplementary material for: How large is the universe of RNA-like motifs? A clustering analysis of RNA graph motifs using topological descriptors
Source: PLoS Comput Biol. 2025 Jul 15;21(7):e1013230. doi: 10.1371/journal.pcbi.1013230 (PMC12262848; doi:10.1371/journal.pcbi.1013230)
Supplement: S1 Methods — This section includes the definition of basic topological concepts (S1.1), persistent spectral graphs (S1.2), clustering algorithms (S1.3), and evaluation metrics (S1.4). (PDF) [file pcbi.1013230.s001.pdf]

Supporting Information: How Large is the Universe of RNA-like Motifs? A Clustering Analysis of RNA Graph Motifs Using Topological Descriptors

Rui Wang [1] and Tamar Schlick* [1,2,3,4]

[1]Simons Center for Computational Physical Chemistry, New York University, New York, NY 10003, USA
[2]Department of Chemistry, New York University, New York, NY 10003, USA
[3]Courant Institute of Mathematical Sciences, New York University, New York, NY 10012, USA
[4]New York University-East China Normal University Center for Computational Chemistry, New York University Shanghai, Shanghai 200122, China

June 29, 2025

Contents

S1 Supplementary Methods **2**
S1.1 Basic topological concepts . . . 2
S1.1.1 Topological concepts . . . 2
S1.1.2 Combinatorial Laplacians . . . 2
S1.2 Persistent spectral graphs . . . 3
S1.3 Clustering Algorithms . . . 4
S1.3.1 k-means clustering . . . 4
S1.3.2 Mini-batch k-means clustering . . . 4
S1.3.3 Gaussian mixture model (GMM) . . . 5
S1.3.4 Hierarchical clustering using Ward's method . . . 5
S1.3.5 Spectral clustering . . . 6
S1.3.6 Birch balanced iterative reducing and clustering using hierarchies) . . . 6
S1.4 Evaluation Metrics . . . 7
S1.4.1 Silhouette score and homogeneity score . . . 7
S1.4.2 Sensitivity of binary clusters . . . 7
S1.4.3 UMAP plots . . . 8
S1.4.4 Hartigan's dip test and Silverman's test . . . 8

S1 Supplementary Methods

S1.1 Basic topological concepts

S1.1.1 Topological concepts

In this section, we give a concise review of simplex, simplicial complex, and chain complex to provide an essential background for persistent spectral graphs. More details can be found in the literature [?, 1]. A *q-simplex* denoted as σ is the convex hull of $q+1$ independent points in coordinate space. For example, a vertex is a 0-simplex, an edge is a 1-simplex, a triangle is a 2-simplex, and a tetrahedron is a 3-simplex. We call the convex hull of each non-empty subset of $q+1$ points a face of σ, and each of its corner points is also called one of its vertices. A set of simplices is a *simplicial complex* K if 1) All faces of any simplex in K are also in K, and the non-empty intersection of any two simplices in K is a common face of the two simplices. A *q-chain* is a formal sum of q-simplices in simplicial complex K with $\mathbb{Z}_2$ coefficients. The set of all q-chains has a basis which the set of q-simplices in K, thus forming a finitely generated free abelian group denoted as $C_q(K)$. The boundary operator is a group homomorphism defined by $\partial_q : C_q(K) \to C_{q-1}(K)$ to relate the chain groups. More specifically, denoting q-simplex as $\sigma_q = [v_0, v_1, \cdots, v_q]$ by its vertices v_i, the boundary operator is defined through its action on the basis,

$$\partial_q \sigma_q = \sum_{i=0}^{q} (-1)^i \sigma_{q-1}^i. \tag{1}$$

Here, $\sigma_{q-1}^i = [v_0, \cdots, \hat{v_i}, \cdots, v_q]$ is the $(q-1)$-simplex with v_i omitted. The following sequence of chain groups connected by boundary operators is a *chain complex* (defined as a set of abelian groups connected by homomorphisms such that the composite of any two consecutive homomorphisms is zero, $\partial_q \partial_{q+1} = 0$.)

$$\cdots \xrightarrow{\partial_{q+2}} C_{q+1}(K) \xrightarrow{\partial_{q+1}} C_q(K) \xrightarrow{\partial_q} C_{q-1}(K) \xrightarrow{\partial_{q-1}} \cdots$$

S1.1.2 Combinatorial Laplacians

A key concept in combinatorial Laplacians is the chain complex associated with a simplicial complex, which is used to define its q-th homology group, given by $H_q = \ker \partial_q / \operatorname{im} \partial_{q+1}$. This homology group is a topological invariant that captures essential features of the simplicial complex. The dimension of H_q, known as the q-th Betti number, $\beta_q = \dim H_q$, measures the number of q-dimensional holes in the simplicial complex, or equivalently, in the geometric object represented by the simplicial complex.

The q-combinatorial Laplacian operator, defined as the linear operator $\Delta_q : C^q(K) \to C^q(K)$,

$$\Delta_q := \partial_{q+1} \partial_{q+1}^* + \partial_q^* \partial_q. \tag{2}$$

Correspondingly, the matrix representation of Δ_q is the qth-order Laplacian, which is denoted $\mathcal{L}_q(K)$,

$$\mathcal{L}_q(K) = \mathcal{B}_{q+1} \mathcal{B}_{q+1}^T + \mathcal{B}_q^T \mathcal{B}_q. \tag{3}$$

Since the qth-order Laplacian $\mathcal{L}_q(K)$ is symmetric and positive semi-definite, its spectrum consists of only real and non-negative eigenvalues. We denote the spectrum of $\mathcal{L}_q(K)$ as

$$\text{Spec}(\mathcal{L}_q(K)) = \{\lambda_{1,q}, \lambda_{2,q}, \cdots, \lambda_{N_q,q}\}.$$

The multiplicity of zero in the spectrum (also called the harmonic spectrum) reveals the topological information β_q, whereas the non-harmonic spectrum encodes further geometric information. The correspondence between the multiplicity of zero spectra of $\mathcal{L}_q(K)$ and the qth Betti number is defined as

$$\beta_q = \dim \ker \partial_q - \dim \operatorname{im} \partial_{q+1} = \dim \ker \mathcal{L}_q(K) = \#0 \text{ eigenvalues of } \mathcal{L}_q(K). \tag{4}$$

Intuitively, β_0 represents the number of connected components in K, β_1 reveals the number of 1D non-contractible loops or circles in K, and β_2 shows the number of 2D voids or cavities in K [2].

S1.2 Persistent spectral graphs

Both topological and geometric information can be derived from analyzing the spectra of qth-order Laplacian. However, the information is restricted to those pieces contained in the connectivity of the simplicial complex. For practical applications, such as feature extraction in machine learning, a single simplicial complex often fails to capture sufficient information. To address this limitation, the Persistent Spectral Graph (PSG) framework is introduced, which generates a sequence of simplicial complexes by varying a filtration parameter, thereby enriching the spectral information available for analysis

First, we consider a filtration of simplicial complex K which is a nested sequence of subcomplexes $(K_t)_{t=0}^m$ of the final complex K:

$$\emptyset = K_0 \subseteq K_1 \subseteq K_2 \subseteq \cdots \subseteq K_m = K. \tag{5}$$

Second, for each subcomplex K_t, we denote its corresponding chain group to be $C_q(K_t)$, and the q-boundary operator will be denoted by $\partial_q^t : C_q(K_t) \to C_{q-1}(K_t)$. As conventionally done, we define $C_q(K_t)$ for $q < 0$ as the zero group $\{0\}$ and ∂_q^t as a zero map. [1] If $0 < q \leq \dim K_t$, then

$$\partial_q^t(\sigma_q) = \sum_i^q (-1)^i \sigma_{q-1}^i, \quad \forall \sigma_q \in K_t, \tag{6}$$

with $\sigma_q = [v_0, \cdots, v_q]$ being any q-simplex, and $\sigma_{q-1}^i = [v_0, \cdots, \hat{v}_i, \cdots, v_q]$ being the $(q-1)$-simplex constructed by removing v_i . The adjoint operator of ∂_q^t is the coboundary operator $\partial_q^{t^*} : C^{q-1}(K_t) \to C^q(K_t)$, which can be regarded as a map from $C_{q-1}(K_t)$ to $C_q(K_t)$ through the isomorphisms $C^q(K_t) \cong C_q(K_t)$ between cochain groups and chain groups.

With this representation, a sequence of chain complexes can be defined as below:

$$\begin{array}{cccccccccccccc}
\cdots & C^1_{q+1} & \underset{\partial^{1^*}_{q+1}}{\overset{\partial^1_{q+1}}{\rightleftharpoons}} & C^1_q & \underset{\partial^{1^*}_{q}}{\overset{\partial^1_{q}}{\rightleftharpoons}} & \cdots & \underset{\partial^{1^*}_{3}}{\overset{\partial^1_{3}}{\rightleftharpoons}} & C^1_2 & \underset{\partial^{1^*}_{2}}{\overset{\partial^1_{2}}{\rightleftharpoons}} & C^1_1 & \underset{\partial^{1^*}_{1}}{\overset{\partial^1_{1}}{\rightleftharpoons}} & C^1_0 & \underset{\partial^{1^*}_{0}}{\overset{\partial^1_{0}}{\rightleftharpoons}} & C^1_{-1} = \{0\} \\
 & \rotatebox{-90}{$\subseteq$} & & \rotatebox{-90}{$\subseteq$} & & & & \rotatebox{-90}{$\subseteq$} & & \rotatebox{-90}{$\subseteq$} & & \rotatebox{-90}{$\subseteq$} & & \\
\cdots & C^2_{q+1} & \underset{\partial^{2^*}_{q+1}}{\overset{\partial^2_{q+1}}{\rightleftharpoons}} & C^2_q & \underset{\partial^{2^*}_{q}}{\overset{\partial^2_{q}}{\rightleftharpoons}} & \cdots & \underset{\partial^{2^*}_{3}}{\overset{\partial^2_{3}}{\rightleftharpoons}} & C^2_2 & \underset{\partial^{2^*}_{2}}{\overset{\partial^2_{2}}{\rightleftharpoons}} & C^2_1 & \underset{\partial^{2^*}_{1}}{\overset{\partial^2_{1}}{\rightleftharpoons}} & C^2_0 & \underset{\partial^{2^*}_{0}}{\overset{\partial^2_{0}}{\rightleftharpoons}} & C^2_{-1} = \{0\} \\
 & \vdots & & \vdots & & & & \vdots & & \vdots & & \vdots & & \vdots \\
 & \rotatebox{-90}{$\subseteq$} & & \rotatebox{-90}{$\subseteq$} & & & & \rotatebox{-90}{$\subseteq$} & & \rotatebox{-90}{$\subseteq$} & & \rotatebox{-90}{$\subseteq$} & & \\
\cdots & C^m_{q+1} & \underset{\partial^{m^*}_{q+1}}{\overset{\partial^m_{q+1}}{\rightleftharpoons}} & C^m_q & \underset{\partial^{m^*}_{q}}{\overset{\partial^m_{q}}{\rightleftharpoons}} & \cdots & \underset{\partial^{m^*}_{3}}{\overset{\partial^m_{3}}{\rightleftharpoons}} & C^m_2 & \underset{\partial^{m^*}_{2}}{\overset{\partial^m_{2}}{\rightleftharpoons}} & C^m_1 & \underset{\partial^{m^*}_{1}}{\overset{\partial^m_{1}}{\rightleftharpoons}} & C^m_0 & \underset{\partial^{m^*}_{0}}{\overset{\partial^m_{0}}{\rightleftharpoons}} & C^m_{-1} = \{0\}
\end{array} \tag{7}$$

For simplicity, C_q^t denotes the chain group $C_q(K_t)$.

Third, we introduce persistence to the Laplacian spectra. We define the subset of C_q^{t+p} whose boundary is in C_{q-1}^t as $\mathbb{C}_q^{t,p}$, assuming the natural inclusion map from C_{q-1}^t to C_{q-1}^{t+p},

$$\mathbb{C}_q^{t,p} := \{\beta \in C_q^{t+p} \mid \partial_q^{t+p}(\beta) \in C_{q-1}^t\}. \tag{8}$$

On this subset, we define the p-persistent q-boundary operator denoted by $\eth_q^{t,p} : \mathbb{C}_q^{t,p} \to C_{q-1}^t$. Its corresponding adjoint operator is $(\eth_q^{t,p})^* : C_{q-1}^t \to \mathbb{C}_q^{t,p}$. We then define the q-order p-persistent Laplacian operator $\Delta_q^{t,p} : C_q^t \to C_q^t$ associated with the filtration as

$$\Delta_q^{t,p} = \eth_{q+1}^{t,p} \left(\eth_{q+1}^{t,p}\right)^* + \partial_q^{t^*} \partial_q^t. \tag{9}$$

[1] We define the boundary matrix $\mathcal{B}_0^t$ for the boundary operator ∂_0^t as a zero matrix. The number of columns of $\mathcal{B}_0^t$ is the number of 0-simplices in K_t, the number of rows will be 1.

The matrix representation of $\Delta_q^{t,p}$ in the simplicial basis is

$$\mathcal{L}_q^{t,p} = \mathcal{B}_{q+1}^{t,p}(\mathcal{B}_{q+1}^{t,p})^T + (\mathcal{B}_q^t)^T\mathcal{B}_q^t, \tag{10}$$

where $\mathcal{B}_{q+1}^{t,p}$ is the matrix representation of $\eth_{q+1}^{t,p}$.

We denote the spectrum of $\mathcal{L}_q^{t,p}$ as

$$\text{Spec}(\mathcal{L}_q^{t,p}) = \{\lambda_{1,q}^{t,p}, \lambda_{2,q}^{t,p}, \cdots, \lambda_{N_q^t,q}^{t,p}\},$$

where $N_q^t = \dim C_q^t$ is the number of q-simplices in K_t, and the eigenvalues are listed in the ascending order. Thus, the smallest non-zero eigenvalue of $\mathcal{L}_q^{t,p}$ is denoted as $\lambda_{2,q}^{t,p}$. We may recognize the multiplicity of zero in the spectrum of $\mathcal{L}_q^{t,p}$ as the qth order p-persistent Betti number $\beta_q^{t,p}$, which counts the number of (independent) q-dimensional holes in K_t that still exists in K_{t+p}. The relation can be observed in

$$\beta_q^{t,p} = \dim\ker\partial_q^t - \dim\operatorname{im}\eth_{q+1}^{t,p} = \dim\ker\mathcal{L}_q^{t,p} = \#0 \text{ eigenvalues of } \mathcal{L}_q^{t,p}. \tag{11}$$

We mainly focus on the $0, 1, 2$th-order persistent Laplacians, which depict the relations among vertices, edges, triangles, and tetrahedra, as we target 3D real-world applications [2,3].

S1.3 Clustering Algorithms

S1.3.1 k-means clustering

The k-means clustering is an unsupervised machine learning method that partitions n data points $\mathbf{x} = \{\mathbf{x}_1, \mathbf{x}_2, \ldots, \mathbf{x}_n\}$ into k clusters $\mathbf{S} = \{S_1, S_2, \ldots, S_k\}$ ($k \leq n$) so as to minimize the within-cluster sum of squares (WCSS). It was first proposed by Stuart Lloyd in 1957 [4]. The objective of k-means clustering is to find:

$$\arg\min_{\mathbf{S}} \sum_{j=1}^{k} \sum_{\mathbf{x}\in S_j} \|\mathbf{x} - \mu_j\|^2, \tag{12}$$

where $\mu_j = \frac{1}{|S_j|}\sum_{\mathbf{x}\in S_j}\mathbf{x}$ is the cluster centroid of points in S_j.

The standard algorithm (naive k-means) works as follows:

1. Initialize k cluster centroids randomly.
2. Assign each data point to its nearest cluster centroid. This will form k clusters.
3. Update the centroids as the mean of the data points in each cluster.
4. Repeat the process and update steps until convergence.

Usually, researchers applied the elbow method [5] to find the optimal number of clusters (k) for k-means clustering. In this work, instead of finding the optimal number of clusters, we aim to separate out graph topologies into exactly two clusters. Therefore, we will not apply the elbow method.

S1.3.2 Mini-batch k-means clustering

Mini-batch k-means clustering that was proposed by D. Sculley in 2010 is a variation of the standard k-means clustering algorithm [6]. The goal of mini-batch k-means clustering is to handle massive datasets efficiently by using small and random subsets (mini-batches) to update the cluster centroids. The algorithm is similar to k-means clustering but only chooses a mini-batch of points instead of all points in step 2 as follows:

1. Initialize k cluster centroids randomly.
2. For each epoch, randomly select a mini-batch of data points and then assign these points to their nearest cluster centroid. This will form k clusters.
3. Update the centroids as the mean of the data points in each cluster.
4. Repeat the process and update steps until convergence.

Mini-batch k-means clustering is good at handling really large datasets to save computational memory and time. However, this algorithm may not have good clustering performance on small datasets.

S1.3.3 Gaussian mixture model (GMM)

The Gaussian mixture model (GMM) is a probabilistic model that assumes the data are generated from a mixture of several Gaussian distributions with unknown parameters. GMM uses the Expectation-Maximization (EM) algorithm to estimate the parameters iteratively. Specifically, for a given dataset $\mathbf{x} = \{\mathbf{x}_1, \mathbf{x}_2, \ldots, \mathbf{x}_n\}$, the GMM assumes that each data point $\mathbf{x}_i$ is generated from a mixture of K Gaussian distributions. Then the probability density function for the GMM is defined as:

$$p(\mathbf{x}_i \mid \Theta) = \sum_{k=1}^{K} \pi_k \mathcal{N}(\mathbf{x}_i \mid \boldsymbol{\mu}_k, \boldsymbol{\Sigma}_k). \quad (13)$$

Here, π_k is the mixing coefficient for the k-th Gaussian component, which satisfies $\sum_{k=1}^{K} \pi_k = 1$ and $\pi_k \geq 0$. $\mathcal{N}(\mathbf{x}_i \mid \boldsymbol{\mu}_k$ is the gaussian probability density function with mean μ_k and covariance $\sum_k$

1. Initialize the parameters (means, covariances, and mixing coefficients) randomly.
2. **Expectation (E) step**: Compute the responsibility that each Gaussian distribution has for each data point.
3. **Maximization (M) step**: Update the parameters based on the current responsibilities.
4. Repeat the E and M steps until convergence, typically when the log-likelihood of the data stops increasing significantly.

We apply GMMs to our datasets to account for the possibility of overlapping clusters with different shapes and sizes.

S1.3.4 Hierarchical clustering using Ward's method

Hierarchical clustering using Ward's method is an agglomerative clustering method that seeks to build a hierarchy of clusters by minimizing the total within-cluster variance [7]. Given a dataset $\mathbf{x} = \{\mathbf{x}_1, \mathbf{x}_2, \ldots, \mathbf{x}_n\}$, the within-cluster variance for a cluster C with mean $\mathbf{m}_C$ is defined as:

$$W(C) = \sum_{\mathbf{x}_i \in C} \|\mathbf{x}_i - \mathbf{m}_C\|^2. \quad (14)$$

For two clusters C_1 and C_2, the increase in total within-cluster variance is defined as:

$$\Delta W = \frac{|C_1| \cdot |C_2|}{|C_1| + |C_2|} \|\mathbf{m}_{C_1} - \mathbf{m}_{C_2}\|^2 \quad (15)$$

Steps of hierarchical ward clustering method are as follows:

1. Consider each data point as a separate cluster and calculate the distance matrix.

2. Merge the two closest clusters that result in the smallest increase in the total within-cluster variance (ΔW).

3. Update the distance matrix to reflect the merge.

4. Repeat the process until only one cluster remains or a predefined number of clusters is achieved.

Notably, the hierarchical (Ward) clustering method is extremely time- and memory-consuming. Therefore, it is slow for even medium-sized datasets. However, this method works effectively even with small datasets when splitting into just two clusters, while some other clustering methods may struggle with smaller samples.

S1.3.5 Spectral clustering

Spectral clustering uses the spectrum of the similarity matrix of the data to perform dimensionality reduction before applying a standard clustering algorithm (e.g., k-means clustering) [8]. Given a set of data points $\mathbf{x} = \{\mathbf{x}_1, \mathbf{x}_2, \ldots, \mathbf{x}_n\}$, we construct a similarity matrix $\mathbf{W} = (w_{ij})$ using radial basis function (RBF) kernel:

$$w_{ij} = \exp\left(-\frac{\|\mathbf{x}_i - \mathbf{x}_j\|^2}{2\sigma^2}\right), \tag{16}$$

where σ is a free parameter. Then, we complete the degree matrix $\mathbf{D}$ (a diagonal matrix) where each diagonal element $d_{ii} = \sum_{j=1}^{N} w_{ij}$. Its corresponding Laplacian matrix can be defined as $\mathbf{L} = \mathbf{D} - \mathbf{W}$. Alternatively, the normalized Laplacian matrix is defined as

$$\mathbf{L}_{\text{norm}} = \mathbf{D}^{-1/2}\mathbf{L}\mathbf{D}^{-1/2} = \mathbf{I} - \mathbf{D}^{-1/2}\mathbf{W}\mathbf{D}^{-1/2} \tag{17}$$

The basic algorithm of spectral clustering is:

1. Construct the similarity matrix $\mathbf{W}$ from the dataset and calculate its Laplacian matrix ($\mathbf{L}$) or normalized Laplacian matrix $\mathbf{L}_{\text{norm}}$.

2. Compute the first k eigenvectors $\mathbf{u}_1, \mathbf{u}_2, \ldots, \mathbf{u}_k$ of the (normalized) Laplacian matrix.

3. Let $\mathbf{U} \in \mathrm{R}^{n \times k}$ to be the reduced-domensional matrix containing the vectors $\mathbf{u}_1, \mathbf{u}_2, \ldots, \mathbf{u}_k$ as columns.

4. Apply a clustering algorithm (e.g., k-means clustering) to the matrix $\mathbf{U}$.

This algorithm works well for capturing the intrinsic geometry of the data, but it is computationally expensive due to the eigenvalue decomposition.

S1.3.6 Birch balanced iterative reducing and clustering using hierarchies)

BIRCH is a hierarchical clustering method designed to efficiently handle particularly large datasets [9]. The BIRCH algorithm follows three steps:

1. **Building the clustering feature (CF) Tree:** The CF tree is a set of entries that summarize information about a cluster of data points. A CF tree of a given dataset $\mathbf{x} = \{\mathbf{x}_1, \mathbf{x}_2, \ldots, \mathbf{x}_n\}$ is defined as

   $$\text{CF} = (n, \text{LS}, \text{SS}), \tag{18}$$

   where LS and SS are the linear sum and the square sum of the data points in the cluster, respectively. They are expressed as:

   $$\text{LS} = \sum_{i=1}^{n} \mathbf{x}_i, \quad \text{SS} = \sum_{i=1}^{n} \mathbf{x}_i^2 \tag{19}$$

2. **Rebuild a smaller CF Tree:** Remove sparse clusters and merge similar clusters to reduce the CF tree size. This step is optional.
3. **Global clustering:** Apply a global clustering algorithm, such as k-means clustering or agglomerative hierarchical clustering algorithm, to cluster all leaf entries of the CF tree to get the final clusters.

BIRCH is suitable to handle large datasets, but it may not perform well with datasets that contain clusters with varying sizes and densities.

S1.4 Evaluation Metrics

S1.4.1 Silhouette score and homogeneity score

The silhouette score and homogeneity score are two widely used metrics to evaluate the effectiveness of clustering algorithms. The silhouette score measures how similar a data point is to its own cluster compared to other clusters. The range of silhouette score is $[-1, 1]$, where a high score indicates that the data point better matches its own cluster and poorly matches the other cluster [10]. The silhouette score for a single data point i is defined as:

$$s(i) = \frac{b(i) - a(i)}{\max(a(i), b(i))}, \tag{20}$$

where $a(i)$ is the mean distance between i and all other data points in the same cluster, and $b(i)$ is the smallest mean distance of i to all points in any other cluster. Here, any distance metric can be used to calculate silhouette scores, such as the Manhattan distance or the Euclidean distance.

The homogeneity score measures whether the clusters are pure concerning the ground truth labels. Assume $C = \{c_1, c_2, \ldots, c_n\}$ is the set of clusters, $K = \{k_1, k_2, \ldots, k_m\}$ is the set of true classes, and the total number of data points is N. Let $A = (a_{ij})$ be the contingency table produced by the clustering algorithm representing the clustering results. Here element a_{ij} shows the number of data points that are in the class k_i and cluster c_j [11]. Then the homogeneity score is defined as

$$h = 1 - \frac{H(K|C)}{H(K)}, \tag{21}$$

where $H(K|C) = -\sum_{c=1}^{|C|}\sum_{k=1}^{|K|} \frac{a_{kc}}{N} \log\left(\frac{a_{kc}}{\sum_{k=1}^{|K|} a_{kc}}\right)$ and $H(K) = -\sum_{k=1}^{|K|} \frac{\sum_{k=1}^{|K|} a_{kc}}{n} \log\left(\frac{\sum_{k=1}^{|K|} a_{kc}}{n}\right)$.

The range of homogeneity score is $[0, 1]$. A homogeneity score of 1 indicates a perfect homogeneity score, or that each cluster contains only members of a single class.

S1.4.2 Sensitivity of binary clusters

Sensitivity evaluates the ability of a clustering method to correctly identify a specific class when there are only two clusters. The definition of sensitivity is

$$\text{Sensitivity} = \frac{\text{TP}}{\text{TP} + \text{FN}}, \tag{22}$$

where TP (true positive) indicates the number of data points that are correctly clustered into the positive class, and FN (false negatives) indicates the number of data points that belong to the positive class but are incorrectly clustered into the negative class. In our work, the graph topologies that match existing RNA structures are defined as the positive class.

Fig A shows the comparison of the silhouette score and the homogeneity score across six clustering methods. It is worth mentioning that the interpretation of these metrics requires careful consideration due to

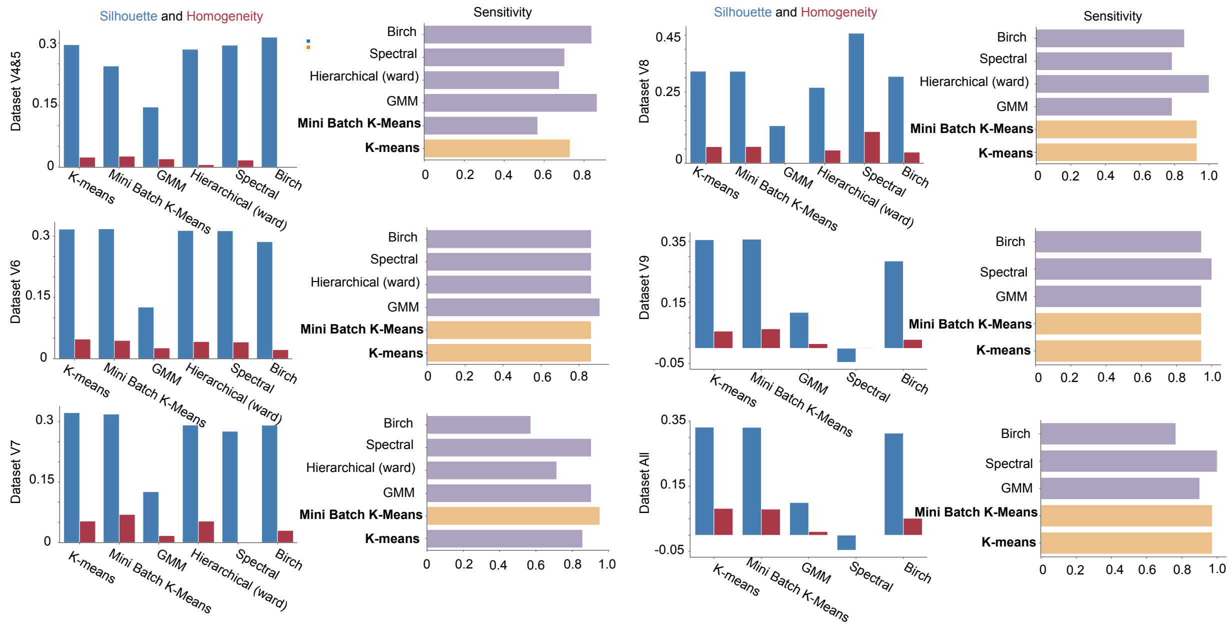

Fig A: Comparison of silhouette score and homogeneity score across six clustering methods. The blue bar represents the silhouette score, while the red bar shows the homogeneity value. The purple and yellow bars represent sensitivities on each dataset across different clustering methods, with yellow indicating the highest and most reliable sensitivity.

our dataset's unique characteristics: our ground truth contains only confirmed RNA structures (label=1), while unlabeled data may include both non-RNA-like structures and undetected RNAs. This deflates both scores because 1) silhouette scores assume well-separated clusters, which doesn't account for potential false negatives in unlabeled data, and 2) homogeneity metrics become unreliable with a single positive class. Therefore, we emphasize comparative performance between methods rather than absolute values. From Fig A, K-means clustering and mini-batch K-means have the best silhouette and homogeneity scores across all datasets.

S1.4.3 UMAP plots

We have incorporated UMAP [12] embeddings as an alternative dimensionality reduction approach to further validate our clustering results across all datasets. Fig B shows umap plots on Dataset V4&5, V6, V7, and V8. Other UMAP plots can be found in the supporting_img folder of PSGRNAClustering. All these UMAP plots show geometric separation between RNA-like and non-RNA-like clusters. The existing RNAs predicted as non-RNA-like are a very small minority in large datasets (Datasets V7, V8, V9, and All), which include a wide range of topologies. In contrast, clustering methods applied to smaller datasets (Datasets V4&5, V6), each containing only 100–500 data samples, exhibit higher misclassification rates. This discrepancy likely arises because distance-based partitioning becomes unstable when the sample size is insufficient to reliably capture the underlying cluster structure, especially when topological differences between clusters are subtle.

S1.4.4 Hartigan's dip test and Silverman's test

In addition, we have performed Hartigan's dip test [13] and Silverman's test [14] to determine whether our data are unimodal or multimodal across all different datasets. Fig C below shows that the 1st feature has a dip value equal to 0.075 with p-value less than 0.05, and the number of peaks is equal to 16, indicating that our data are multimodal and therefore the 50% RNA-like is not from the K-means applied to a unimodal distribution. The similar dip distribution and density estimation plots for other features can be found in the peak_detection folder of a github repo PSGRNAClustering.

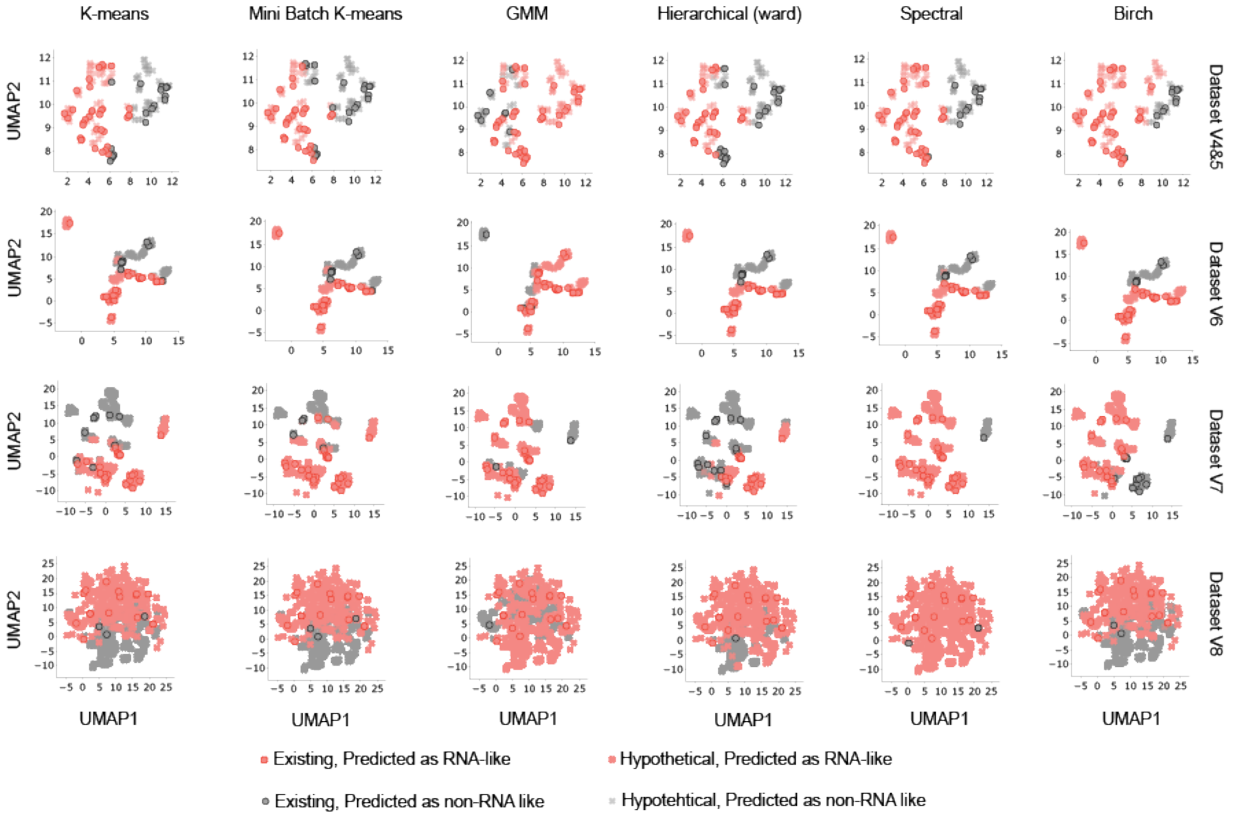

Fig B: Clustering results by six methods with persistent spectral graph-based features on six datasets grouped by motif vertices: Dataset V4&5, Dataset V6, Dataset V7, and Dataset V8. The red and grey clusters are the RNA-like and non-RNA-like clusters, respectively. The circled red dot symbols indicate existing RNA molecules, while the cross symbols denote hypothetical graphs. The first two methods (k-means) exhibit the most reliable performance.

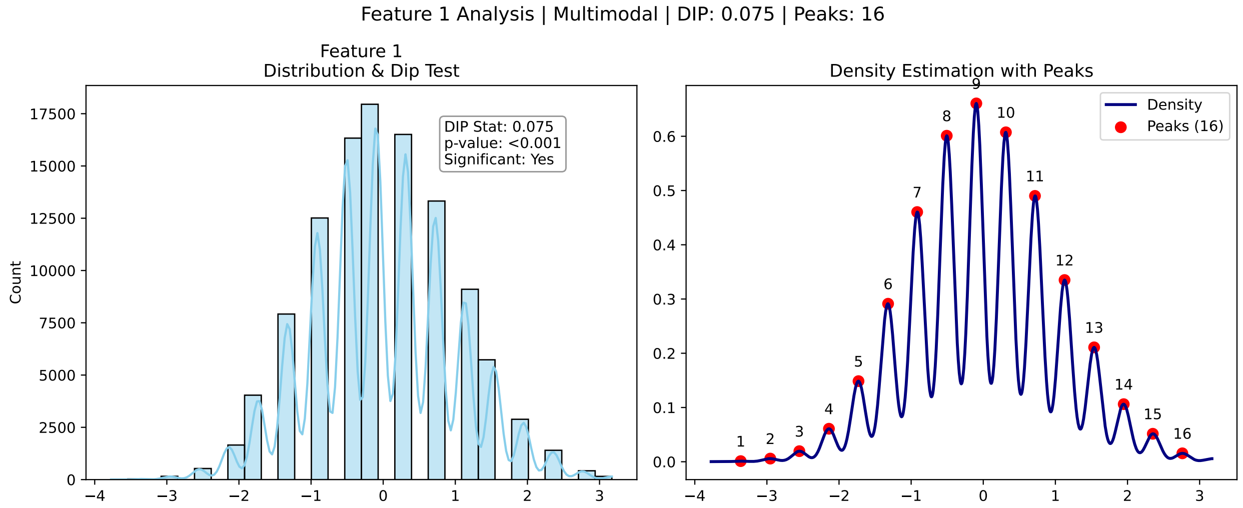

Fig C: The Hartigan's dip test and Silverman's test results on the 1st feature from the Dataset All. The dip value is 0.075 with a p-value less than 0.05, and the number of estimated peaks is equal to 16.

References

[1] Edelsbrunner H, Harer J, et al. Persistent homology-a survey. Contemporary Mathematics. 2008;453:257-82.

[2] Wang R, Zhao R, Ribando-Gros E, Chen J, Tong Y, Wei GW. HERMES: Persistent spectral graph software. Foundations of Data Science. 2021;3(1):67.

[3] Wang R, Nguyen DD, Wei GW. Persistent spectral graph. International Journal for Numerical Methods in Biomedical Engineering. 2020;36(9):e3376.

[4] Lloyd S. Least squares quantization in PCM. IEEE Transactions on Information Theory. 1982;28(2):129-37.

[5] Bholowalia P, Kumar A. EBK-means: A clustering technique based on elbow method and k-means in WSN. International Journal of Computer Applications. 2014;105(9).

[6] Sculley D. Web-scale k-means clustering. In: Proceedings of the 19th International Conference on World Wide Web. WWW '10. New York, NY, USA: Association for Computing Machinery; 2010. p. 1177–1178. Available from: https://doi.org/10.1145/1772690.1772862.

[7] Ward Jr JH. Hierarchical grouping to optimize an objective function. Journal of the American Statistical Association. 1963;58(301):236-44.

[8] Zare H, Shooshtari P, Gupta A, Brinkman RR. Data reduction for spectral clustering to analyze high throughput flow cytometry data. BMC Bioinformatics. 2010;11:1-16.

[9] Zhang T, Ramakrishnan R, Livny M. BIRCH: an efficient data clustering method for very large databases. ACM Sigmod Record. 1996;25(2):103-14.

[10] Rousseeuw PJ. Silhouettes: a graphical aid to the interpretation and validation of cluster analysis. Journal of Computational and Applied Mathematics. 1987;20:53-65.

[11] Rosenberg A, Hirschberg J. V-measure: A conditional entropy-based external cluster evaluation measure. In: Proceedings of the 2007 joint conference on empirical methods in natural language processing and computational natural language learning (EMNLP-CoNLL); 2007. p. 410-20.

[12] McInnes L, Healy J, Melville J. Umap: Uniform manifold approximation and projection for dimension reduction. arXiv preprint arXiv:180203426. 2018.

[13] Hartigan JA, Hartigan PM. The dip test of unimodality. The annals of Statistics. 1985:70-84.

[14] Hall P, York M. On the calibration of Silverman's test for multimodality. Statistica Sinica. 2001:515-36.
