# Supplementary material for: How large is the universe of RNA-like motifs? A clustering analysis of RNA graph motifs using topological descriptors
Source: PLoS Comput Biol. 2025 Jul 15;21(7):e1013230. doi: 10.1371/journal.pcbi.1013230 (PMC12262848; doi:10.1371/journal.pcbi.1013230)
Supplement: Attachment — Submitted filename: ReplyReviewers.pdf [file pcbi.1013230.s002.pdf]

Authors’ Response to Reviews of: How Large is the Universe of RNA-Like Motifs? A Clustering Analysis of RNA Graph Motifs Using Topological Descriptors (Manuscript ID: COMPBIOL-D-25-00058)

Rui Wang [1] and Tamar Schlick* [1,2,3,4]

[1]Simons Center for Computational Physical Chemistry, New York University, New York, NY 10003, USA
[2]Department of Chemistry, New York University, New York, NY 10003, USA
[3]Courant Institute of Mathematical Sciences, New York University, New York, NY 10012, USA
[4]New York University-East China Normal University Center for Computational Chemistry, New York University Shanghai, Shanghai 200122, China

Dear Editor and Reviewers:

Thank you very much for your mail of 17-Mar-2025, and referees’ reports concerning our manuscript entitled: “How Large is the Universe of RNA-Like Motifs? A Clustering Analysis of RNA Graph Motifs Using Topological Descriptors” (Manuscript ID: PCOMPBIOL-D-25-00058). These comments are all valuable and very helpful for revising and improving our paper, as well as providing important guidance to our researchers. We have accordingly gone through the referees’ comments and have revised the manuscript. In the revised manuscript, the deleted text is marked in red with a strikethrough, while added text is shown in blue with a wavy underline. Full responses to reviewers’ comments are below.

Journal Requirements:

1. Please ensure that the CRediT author contributions listed for every co-author are completed accurately and in full. At this stage, the following Authors/Authors require contributions: Rui Wang. Please ensure that the full contributions of each author are acknowledged in the ”Add/Edit/Remove Authors” section of our submission form. The list of CRediT author contributions may be found here.
2. We ask that a manuscript source file is provided at Revision. Please upload your manuscript file as a .doc, .docx, .rtf or .tex. If you are providing a .tex file, please upload it under the item type u2018LaTeX Source Fileu2019 and leave your .pdf version as the item type u2018Manuscriptu2019.
3. Please provide an Author Summary. This should appear in your manuscript between the Abstract (if applicable) and the Introduction, and should be 150-200 words long. The aim should be to make your findings accessible to a wide audience that includes both scientists and non-scientists. Sample summaries can be found on our website under Submission Guidelines
4. Please upload all main figures as separate Figure files in .tif or .eps format. For more information about how to convert and format your figure files please see our guidelines.
5. Thank you for stating ”The code and data for the feature and clustering algorithms are available at the

public repository PSGRNA-Clustering. The RNA inverse folding using dual graph representations package is available at Dual-RAG-IF." Please provide direct links in the online submission form to access the datasets.

6. Please amend your detailed Financial Disclosure statement. This is published with the article. It must therefore be completed in full sentences and contain the exact wording you wish to be published.

7. State what role the funders took in the study. If the funders had no role in your study, please state: "The funders had no role in study design, data collection and analysis, decision to publish, or preparation of the manuscript."

   Answer: Thanks for the detailed information. We have fulfilled all these journal requirements.

Reviewer 1:

Comments: This work explores the space of all possible dual graphs (a coarse-grained representation of RNA structures) with number of vertices ranging from 4 to 9. These structures are clustered into two groups which are later labeled as RNA-like or not-RNA-like using a handful of known RNA structures. Several commonly used clustering algorithms were tried using statistical features generated from the eigenvalues of the graph Laplacians on a sequence of subgraphs (thresholded by edge weights). The study suggests that 46% of the graphs could correspond to RNA structures. It also provides a landscape for designing novel RNA motifs. I mainly have questions on the (1) reliability of the main conclusion and (2) utilizing the problem setup (small number of features used with interpretable methods) to include interpretations of the model.

1. According to the description in 2.2.1, it seems that "persistence" is never used. A simpler description without unnecessary jargon would make it more readable, especially for readers who are not familiar with the concept of persistence. For example, "exploring the eigenvalues of combinatorial Laplacians at different frames of a filtration."

   Answer: We thank the reviewer for the suggestions. We have elaborated upon persistence in Section 2.2.1 to retain that we are working on 0-persistence Laplacians (i.e., graph Laplacians) at different frames of filtrations. However, we keep the p-persistence term as readers may be interested in trying other non-zero persistence values that are suitable for their own datasets.

2. Related to the previous point, there is also no need to include the parameter q and qth-order persistent Laplacian, as only standard graph Laplacians are used.

   Answer: We appreciate the suggestion from the reviewer. In our results section, we did analyze the Betti 1 feature obtained from the higher-order Laplacians (See Section 3.3).

3. The interpretation of the main result could use more clarification. I have the following questions: (1) It is hard to tell from the scatter plots in Figure 3 whether there is separation between the two clusters. The method of choice, K-means, works well on datasets with clear separation between clusters. For example, if the dataset is drawn from a 1D Gaussian, k-means with k=2 will just divide the dataset into two, one below the mean and one above the mean. The 50% RNA-like and the significantly different percentage from GMM makes me wonder if the 50% is due to applying K-means to a unimodal distribution. It would be helpful to also visualize the distribution as density plots and potentially in different embeddings, such as UMAP, etc. (2) Conceptually, it is possible for the ground-truth to be, for example, 20% of RNA-like structures. However, the current validation can not distinguish between the two (vs. the predicted 46%). It would be helpful to include the additional metrics (like homogeneity scores) in the performance table in the main manuscript for more comprehensive assessment of the performance. Also, is there any resource, direct or indirect, on known negative samples?

If such results exist, including specificity could be very helpful to clarify this point. In addition, if some negative samples are available, it is also interesting to explore semi-supervised learning, which may outperform the current approach where the separation of data and label assignment are done in two sequential steps.

Answer:

(1) *Thanks very much for your insightful comments and specific suggestions.*

We have incorporated UMAP [1] embeddings as an alternative dimensionality reduction approach to further validate our clustering results across all datasets. UMAP embeddings are low-dimensional representations learned by Uniform Manifold Approximation and Projection (UMAP), a nonlinear dimensionality reduction technique that preserves both local and global data structure. Figure 1 below shows UMAP plots on Dataset V4&5, V6, V7, and V8. Other UMAP plots can be found in the supporting_img folder of a github repo PSGRNAClustering. All these UMAP plots show geometric separation between RNA-like and non-RNA-like clusters. The existing RNAs predicted as non-RNA-like are a very small minority in large datasets (Datasets V7, V8, V9, and All), which include a wide range of topologies. In contrast, clustering methods applied to smaller datasets (Datasets V4&5, V6), each containing only 100–500 samples, exhibit higher misclassification rates. This discrepancy likely arises because distance-based partitioning becomes unstable when the sample size is insufficient to reliably capture the underlying cluster structure, especially when topological differences between clusters are subtle.

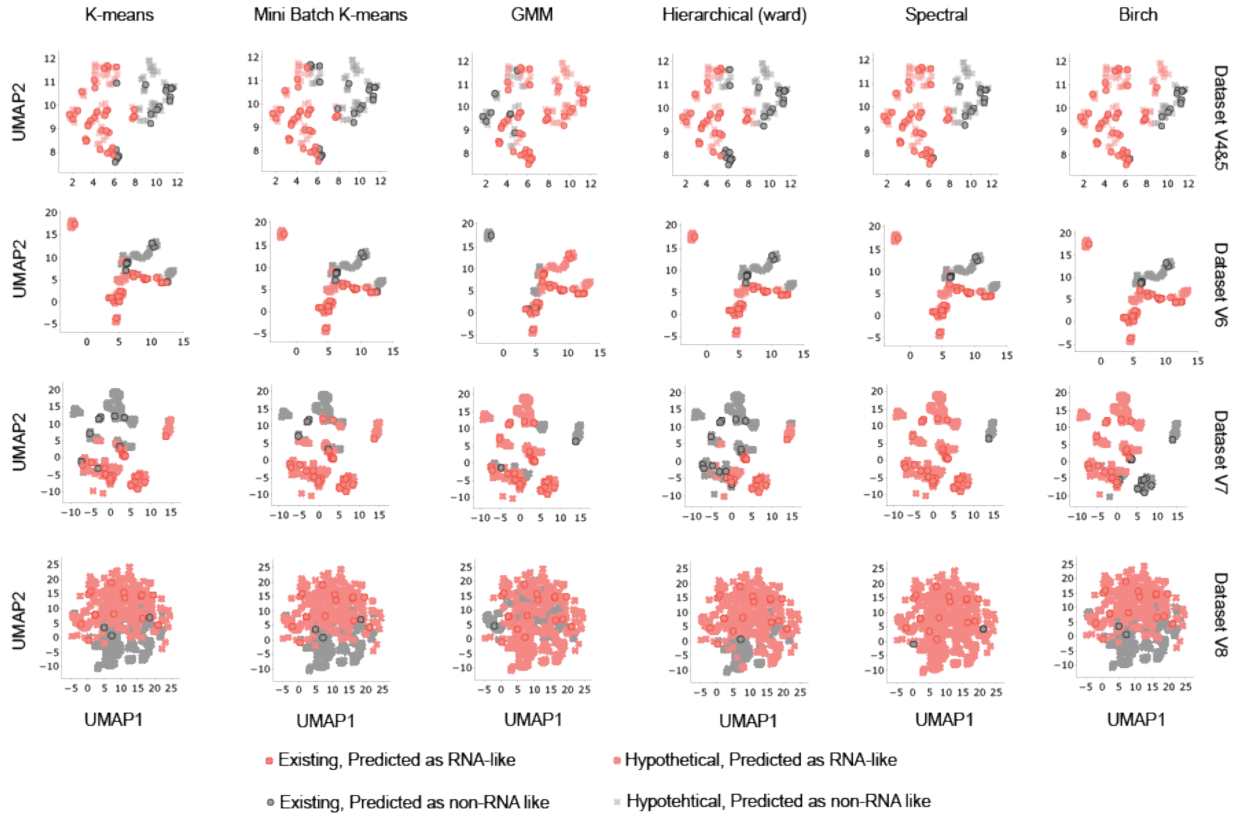

Figure 1: Clustering results by six methods with persistent spectral graph-based features on six datasets grouped by motif vertices: Dataset V4&5, Dataset V6, Dataset V7, and Dataset V8. The red and grey clusters are the RNA-like and non-RNA-like clusters, respectively. The circled red dot symbols indicate existing RNA molecules, while the cross symbols denote hypothetical graphs. The first two methods (k-means) exhibit the most reliable performance.

Moreover, we did report the silhouette and homogeneity score in the Supporting Information. However, the interpretation of these metrics requires careful consideration due to our dataset's unique characteristics:

our ground truth contains only confirmed RNA structures (label=1), while unlabeled data may include both non-RNA structures and undetected RNAs. This deflates both scores because 1) Silhouette scores assume well-separated clusters, which doesn't account for potential false negatives in unlabeled data, and 2) Homogeneity metrics become unreliable with a single positive class. Therefore, we emphasize comparative performance between methods rather than absolute values. From Figure S1 in the Supporting Information, K-means clustering has the best silhouette and homogeneity score across all datasets.

In addition, we have performed Hartigan's dip test [2] and Silverman's test [3] to determine whether our data are unimodal or multimodal across all different datasets. Figure 2 below shows that the 1st feature has a dip value equal to 0.075 with p-value less than 0.05, and the number of peaks is equal to 16, indicating that our data are multimodal and therefore the 50% RNA-like result stems not from the k-means application to a unimodal distribution. The similar dip distribution and density estimation plots for other features can be found in the peak_detection folder of a github repo PSGRNAClustering. We have also added this discussion of new figures to Section 3.1.2 in the revised manuscript.

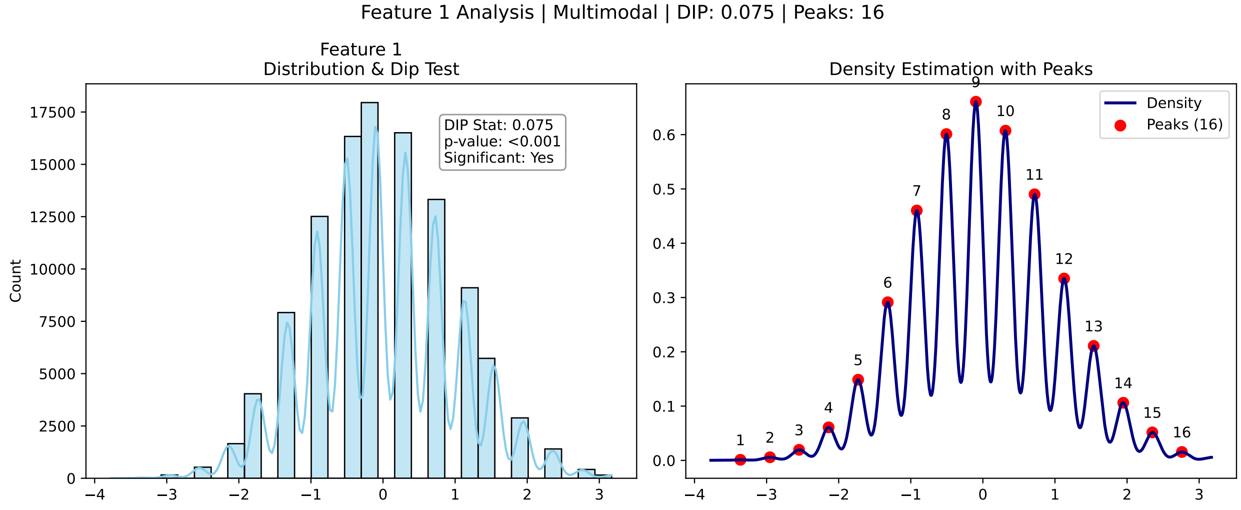

Figure 2: The Hartigan's dip test and Silverman's test results on the 1st feature from the Dataset All. The dip value is 0.075 with a p-value less than 0.05, and the number of estimated peaks is equal to 16.

(2) *Thanks for the thoughtful idea. However, our datasets only have one ground truth label (i.e., graphs corresponding to existing RNAs). No negative samples are available, so any semi-supervised learning methods are not applicable for us.*

4. I find the interpretation in 3.3 interesting. Why is Betti-1 used here but not in the clustering analysis (2.2.1)? It would be interesting to see whether the patterns identified from a few example graphs (Figure 4) apply to the entire dataset. Can you visualize the average Betti curves across all graphs of each cluster? Some statistical tests for distinguishing the curves from the two groups are useful to formally state the observation. Additionally, interpretations of the 18 features should be included and presented in a similar manner.

   Answer: Among all 121 existing RNA graphs collected in 2021, the number of zero value betti-1 and betti-2 can be found in Table 1 below. Because more than 94% Betti-1 and Betti-2 values are zeros, adding them as features will make the performance worse.

   Among all 121 RNA graphs analyzed (collected in 2021), the majority exhibit trivial topological complexity in higher dimensions: as shown in Table 1 below, over 94% of these graphs have zero values for both Betti-1 and Betti-2. This strong prevalence of zero values implies that incorporating Betti-1 and Betti-2 as features would likely introduce noise rather than a meaningful signal, potentially compromising model performance. Therefore,

we did not use Betti-1 in the clustering analysis.

Table 1: The number of zero value Betti-1 and Betti-2 at different filtration parameters.

	Filtration 1	Filtration 2	Filtration 3
Betti-1	110	104	104
Betti-2	121	121	121

Following your suggestions, we include plots reporting average betti number curves along different filtration parameters, as shown in Figure 3 below. From Figure 3 a) and d), we can observe that the average betti-1 along all filtration is less than 1 on existing RNA topologies collected in 2021 and 2022. This indicates that existing RNAs do not tend to form loops on all filtrations. Moreover, although the average betti-1 on the predicted as RNA-like graphs Figure 3 b) is higher than the existing RNA topoglies, we can still observe a significant difference between predicted as RNA-like and predicted as non-RNA-like graphs. The graph predicted as non-RNA-like tend to form more loops compared to RNA-like graphs. This is in line with the statement in Figure 4 in the manuscript.

We now incorporate plots describing average Betti number curves along various filtration parameters (see Section 3.3), as illustrated in Figure 3. From Figure 3 a) and d), we can see that the average Betti-1 value remains below 1 for all existing RNA topologies collected in 2021 and 2022. This observation suggests that existing RNAs are not prone to forming loops under all filtration conditions. Furthermore, although the average Betti-1 value for the predicted RNA-like graphs in Figure Figure 3 b) exceeds that of the existing RNA topologies, we can still notice a substantial difference between the predicted RNA-like and predicted non-RNA-like graphs. Predicted non-RNA-like graphs exhibit a higher tendency to form loops compared to RNA-like graphs, which aligns with the findings presented in Figure 4 of the manuscript. Furthermore, similar Betti-1 patterns exist on top RNA-like (see Figure 3e)) and top non-RNA-like graphs: top RNA-like graphs (see Figure 3 f)) have a relatively lower average Betti-1 number compared to top non-RNA-like graphs.

5. Can the authors verify if the subpanels of Figure 3 are correct? At least for the top left panel, it looks like much more than 139 points (total possible graphs for V4&5).

   Answer: Thanks for pointing this out. We originally misplaced the figure of Dataset V6 on the top left panel. We have updated Figure 3.

6. 2.2.1 states 18 features while 3.1.1 states 19 features.

   Answer: We thank the reviewer for pointing this out. Section 2.2.1 states 18 statistical values of the Spec$(L_q^{d,0})$ *features plus an additional average degree feature. Therefore, in total, we have 19 features. We have clarified in Section 2.2.1 to avoid any future confusion.*

7. I appreciate the property summary of dual graphs in 2.1.1. Can you elaborate more on how the listed properties (non-surjective, non-injective) affect the implication of the clustering analysis results?

   Answer: The **non-surjectivity** *shows that not all possible dual nodes have a corresponding feature in the original RNA graph, especially in sparsely connected or highly irregular secondary structures. This filters out regions with insufficient topological signal and helps us focus more on core architectural elements of RNA that contribute most to structural integrity or functional folding patterns. In practice, this means that clustering analysis operates on a constrained manifold of biologically feasible graphs. In addition, the* **non-injectivity** *of this dual graph setting indicates that distinct RNA secondary structures with different loop, bulge size, or junctions, may map to the same dual graph. This is in the same spirit as the coarse-graining strategy is frequently used in computational biology/chemistry. The detailed geometric or sequence-level distinctions are collapsed into a topological abstraction. As a result, clustering in this dual graph space focuses on higher-order architectural patterns instead of fine-grained structural differences. For example, multiple RNA motifs with different bulge lengths may still cluster together because they share the same stem-loop connectivity at the*

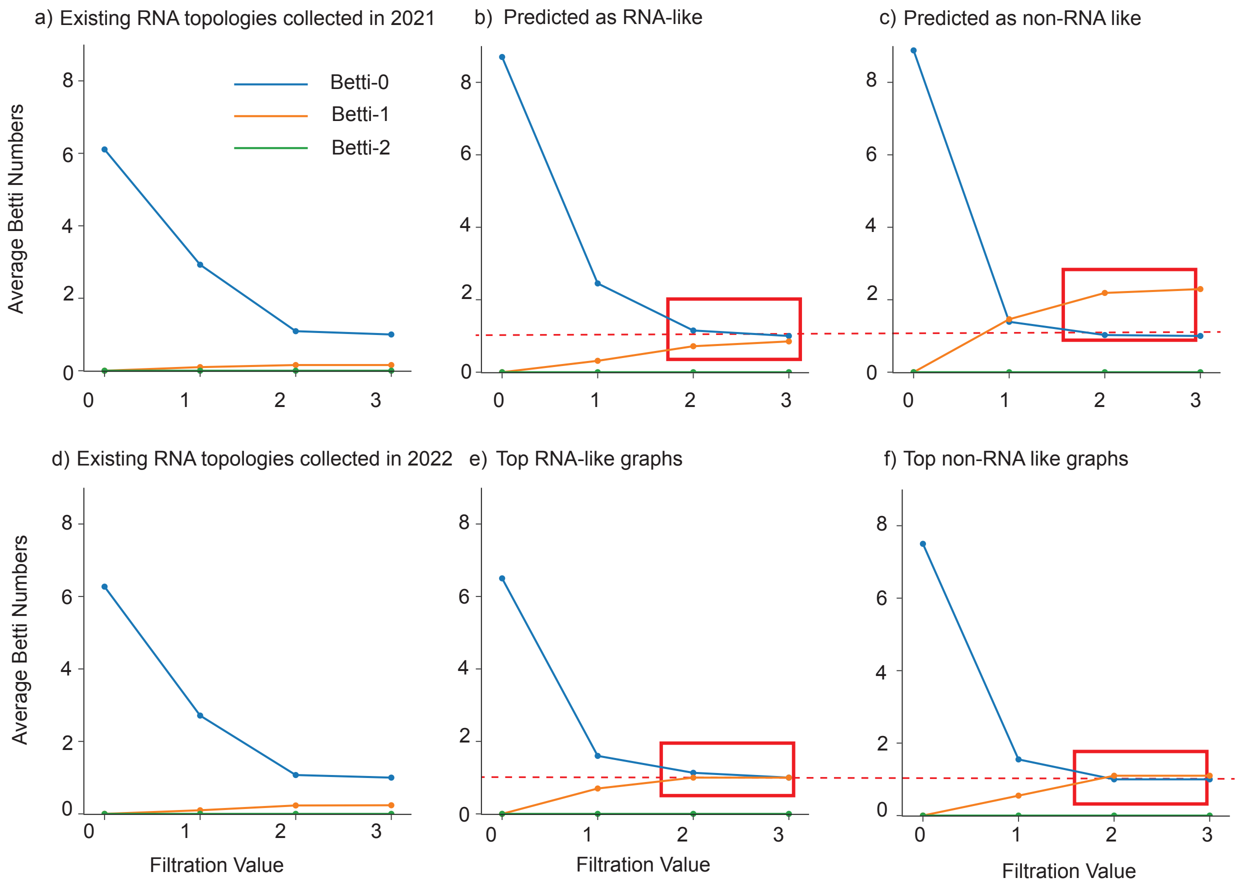

Figure 3: Average Betti number curves along different filtration parameters using data from a) previously collected existing RNA topologies in 2021, b) predicted RNA-like graphs, c) predicted non-RNA-like graphs, d) previously collected RNA topologies in 2022, e) top RNA-like graphs, and f) top non-RNA-like graphs. The blue, orange, and green curves correspond to Betti-0, Betti-1, and Betti-2, respectively.

graph level. While differences in base pairing numbers per stem, for example, are not distinguished, patterns in topology (e.g., stem loop features) can be discerned.

8. I am curious about why the performance is generally worse on smaller graphs? Could including higher-order Laplacians lead to improvements?

   Answer: The performance of machine learning algorithms tends to be poorer on smaller graphs compared to larger graphs. This is because smaller graphs share more similar topological and geometric features, which can lead to reduced performance. We have tried including higher-order Laplacian features, but they do not lead to improvement. The main reason is that almost all Betti-1 and Betti-2 features are zero (see Table 1), while only a few are non-zero. These highly skewed feature distributions have negative impacts on the performance of machine learning algorithms.

   Thank you for your detailed and insightful comments!

Reviewer 2:

This study employs clustering analysis using graph data mining techniques to represent RNA structures through an ”RNA-Like” graph-topological framework. However, several concerns arise:

1. Graph Representation of RNA Structures

A. The authors treat RNA structures as graphs, with double-stranded helical stem regions represented as nodes. However, considering all base-pairing regions as equivalent vertices may introduce bias in the graphical representation of RNA structures. For instance, a short 5-nt stem-loop structure may have a completely different function from a 100-nt long double helix in living cells. Yet, both structures could share an identical graphical representation under this approach, potentially oversimplifying key structural distinctions.

Answer: Thank you for your comments. Yes, of course, we have used a coarse-grained approach that accounts for topological features rather than base-pair details. Such coarse-graining is advantageous for classifying analysis, partitioning, and designing RNAs on a higher level. Thus clearly, the application used with RAG should be chosen appropriately. General advantages are:

a) Dimensionality Reduction: Table 1 in the manuscript lists the numbers of all possible 2D graphs. As the number of vertices increases, the number of possible topologies grows exponentially. With the number of vertices ranging from 2 to 9, there are already 110,667 possible graph topologies available. Introducing stem length as an additional parameter/consideration would require substantially increasing the vertex count, leading to a combinatorial explosion that would make systematic analysis impractical. This coarse-grained method is similar to the strategies in protein or genome studies, where researchers analyze secondary structure topology before optimizing side-chains, or nucleosome-level genomes. Coarse-graining can be exploited for conformational sampling, structure prediction, and mutation design as discussed and applied in our papers (see for example [4])

b) Topological Thinking: Topology usually determines foldability, while stem length fine-tunes functions. For example, although tRNAs and riboswitches have different biological functions, their secondary structures can share identical dual graph topologies. This emphasizes the coarse-grained nature of our representation, where diverse structural RNAs may converge onto a shared topological motif despite substantial biochemical and functional differences. After identifying promising RNA-like topologies, we can apply inverse folding techniques (Section 3.5) to design specific stem lengths and sequences.

Clearly, future refinements, such as weighting nodes by stem length or integrating functional annotations, could further enhance the model's performance. We have added a discussion of these potential extensions in Section 4.2.

B. It is commendable that the authors categorized graph topologies into three groups: existing, RNA-Like, and non-RNA-Like, based partly on prior structural knowledge. However, RNA structures exhibit high dynamic variability. Less frequently observed conformations may be misclassified as non-RNA-Like due to overfitting to a limited existing dataset. Additionally, current RNA secondary and tertiary structure prediction tools have inherent technical limitations, which may further affect the accuracy of the graphical representation. Clarifying these limitations in the manuscript is important.

Answer: We thank the reviewer for this valuable comment. We fully agree that the current RNA secondary and tertiary structure prediction tools have inherent technical limitations that may further affect the accuracy of the graphical representation. That is why in fact we frequently update our classifications and correct any database errors that have occurred over the past two decades.

Our approach has evolved significantly since the initial development of RNA-as-Graph (RAG) in 2003, where we first introduced tree-graph representations for RNA motifs and dual graphs for pseudoknots [5]. As structural data increased, we significantly enhanced the RAG resource in 2011 by incorporating additional RNA structures and developing new analytical tools for substructure identification and graph searching [6]. In the year 2014, we have two important advances: 1) we developed a tree graph partitioning using Fiedler vectors for the discovery of RNA modularity [7], and 2) we introduced RAG 3D tree graphs

to characterize global helical arrangements in large RNA structures [8]. Building on these developments, we expanded the RAG framework in 2015 to handle larger tree graphs (up to 13 vertices, 260 nucleotides) while implementing Partitioning Around Medoids clustering to better classify "RNA-like" motifs from hypothetical structures [9]. In the same year, we also presented RAG-3D, a dataset of RNA tertiary (3D) structures and substructures with a web-based search tool, to enable comprehensive graph-based searches of RNA tertiary structures and substructures [10].

In 2019, we developed enhanced dual graph enumeration algorithms capable of generating complete libraries of dual graph topologies for 2-9 vertices, along with an improved algorithm for RNA subgraph identification [11]. Most recently, in 2021, we incorporated Fiedler vector-based feature design and scoring models to further refine our graph candidate selection process [12].

Our updated approaches have addressed many challenges in RNA structural classification while maintaining computational efficiency. Because coarse-grained graph representations are not suitable for capturing all-atom conformational and dynamic fluctuations important for RNA in vivo, all-atom modeling is essential for many applications (e.g., Yan & Schlick, PNAS 2025 [4], Yan et al., Nature Comm 2023 [13]) to gain a full understanding. Ultimately, experimental validations are needed [14,15].

We fully recognize the inherent complexity and dynamic nature of RNA structural space, and our framework has been designed to accommodate new structural data and prediction tools as they become available. We have expanded our discussion of these considerations in the revised manuscript (Section 4.2)

1. Distinction Between 'RNA-Like' and 'Non-RNA-Like' Graph Classes

   A. While the authors discuss that biological RNA topologies are more likely to contain distinguishing subgraphs, a more comprehensive explanation and comparison between RNA-like and non-RNA-like graph classes is necessary. This should incorporate insights from clustering, persistent subgraph generation (PSG), or other data mining approaches.

   Answer: Thanks for the suggestions. We have added more data mining approaches, such as UMAP [1], Hartigan's dip test [2], and Silverman's test [3] in Section 3.1.2 of the manuscript. In addition, we have included the average Betti curves analysis in Section 3.3. 1) The UMAP plots show geometric separation between RNA-like and non-RNA-like clusters. This provides compelling evidence for the robustness of our clustering approach. This separation is reproduced across two distinct dimensionality reduction methods (UMAP and PCA), with both techniques yielding similar clustering patterns in their respective 2D embeddings. 2) The Hartigan's dip test and Silverman's test results (see Figure 2 below) show that our clustering models apply to a multimodal distribution and therefore the 50% RNA-like is not from the K-means applied to a unimodal distribution. 3) We also include plots describing average Betti number curves along various filtration parameters (see Figure 3 below). These plots show that existing RNAs are not prone to forming loops under all filtration conditions. Moreover, although the average Betti-1 value for the predicted RNA-like graphs in Figure Figure 3 b) below exceeds that of the existing RNA topologies, we still notice a substantial difference between the predicted RNA-like and predicted non-RNA-like graphs. Predicted non-RNA-like graphs exhibit a higher tendency to form loops compared to RNA-like graphs, which aligns with the findings presented in Figure 4 of the manuscript. Furthermore, similar Betti-1 patterns exist on top RNA-like (see Figure 3e) below) and top non-RNA-like graphs: top RNA-like graphs (see Figure 3 f) below) have a relatively lower average Betti-1 number compared to top non-RNA-like graphs.

   B. *If persistent Betti numbers (Betti 0 and Betti 1) can be mapped back to specific biological or structural scenarios, the interpretation will be more intuitive. For instance, if one category consistently retains more connected components, this should be explicitly analyzed and explained.*

   Answer: We appreciate these suggestions from the reviewer. In our methodology, persistent Betti num-

bers are computed through a filtration process applied to RNA dual graphs, as described in Methods 2.3. This approach generates a series of intermediate graphs with evolving connectivity, where the calculated persistent Betti numbers reflect topological changes across filtration steps rather than static features of the original RNA structure. While this dynamic perspective provides valuable information about topological robustness, it does present challenges for direct mapping back to specific structural elements of the native RNA motif.

References

[1] Leland McInnes, John Healy, and James Melville. Umap: Uniform manifold approximation and projection for dimension reduction. *arXiv preprint arXiv:1802.03426*, 2018.

[2] John A Hartigan and Pamela M Hartigan. The dip test of unimodality. *The annals of Statistics*, pages 70–84, 1985.

[3] Peter Hall and Matthew York. On the calibration of Silverman's test for multimodality. *Statistica Sinica*, pages 515–536, 2001.

[4] Shuting Yan and Tamar Schlick. Heterogeneous and multiple conformational transition pathways between pseudoknots of the SARS-CoV-2 frameshift element. *Proceedings of the National Academy of Sciences*, 122(4):e2417479122, 2025.

[5] Hin Hark Gan, Samuela Pasquali, and Tamar Schlick. Exploring the repertoire of RNA secondary motifs using graph theory; implications for RNA design. *Nucleic Acids Research*, 31(11):2926–2943, 2003.

[6] Christian Laing, Dongrong Wen, Jason TL Wang, and Tamar Schlick. Predicting coaxial helical stacking in RNA junctions. *Nucleic acids research*, 40(2):487–498, 2012.

[7] Namhee Kim, Zhe Zheng, Shereef Elmetwaly, and Tamar Schlick. RNA graph partitioning for the discovery of RNA modularity: a novel application of graph partition algorithm to biology. *PlOS One*, 9(9):e106074, 2014.

[8] Namhee Kim, Christian Laing, Shereef Elmetwaly, Segun Jung, Jeremy Curuksu, and Tamar Schlick. Graph-based sampling for approximating global helical topologies of RNA. *Proceedings of the National Academy of Sciences*, 111(11):4079–4084, 2014.

[9] Naoto Baba, Shereef Elmetwaly, Namhee Kim, and Tamar Schlick. Predicting large RNA-like topologies by a knowledge-based clustering approach. *Journal of molecular biology*, 428(5):811–821, 2016.

[10] Mai Zahran, Cigdem Sevim Bayrak, Shereef Elmetwaly, and Tamar Schlick. RAG-3D: a search tool for RNA 3D substructures. *Nucleic Acids Research*, 43(19):9474–9488, 2015.

[11] Swati Jain, Sera Saju, Louis Petingi, and Tamar Schlick. An extended dual graph library and partitioning algorithm applicable to pseudoknotted RNA structures. *Methods*, 162:74–84, 2019.

[12] Qiyao Zhu and Tamar Schlick. A fiedler vector scoring approach for novel RNA motif selection. *The Journal of Physical Chemistry B*, 125(4):1144–1155, 2021.

[13] Shuting Yan, Qiyao Zhu, Jenna Hohl, Alex Dong, and Tamar Schlick. Evolution of coronavirus frameshifting elements: Competing stem networks explain conservation and variability. *Proceedings of the National Academy of Sciences*, 120(20):e2221324120, 2023.

[14] Abhishek Dey, Shuting Yan, Tamar Schlick, and Alain Laederach. Abolished frameshifting for predicted structure-stabilizing SARS-CoV-2 mutants: Implications to alternative conformations and their statistical structural analyses. *RNA*, 30(11):1437–1450, 2024.

[15] Tamar Schlick, Qiyao Zhu, Swati Jain, and Shuting Yan. Structure-altering mutations of the SARS-CoV-2 frameshifting RNA element. *Biophysical Journal*, 120(6):1040–1053, 2021.
